# Self-Harm Thoughts Postpartum as a Marker for Long-Term Morbidity

**DOI:** 10.3389/fpubh.2018.00034

**Published:** 2018-02-19

**Authors:** Stavros I. Iliadis, Alkistis Skalkidou, Hanna Ranstrand, Marios K. Georgakis, Cathrine Axfors, Fotios C. Papadopoulos

**Affiliations:** ^1^Department for Women’s and Children’s Health, Uppsala University, Uppsala, Sweden; ^2^Department of Hygiene, Epidemiology and Medical Statistics, Medical School, National and Kapodistrian University of Athens, Athens, Greece; ^3^Department of Neuroscience, Psychiatry, Uppsala University, Uppsala, Sweden

**Keywords:** self-harm, postpartum depression, maternal morbidity, pregnancy, postpartum period, psychiatric morbidity

## Abstract

**Introduction:**

Postpartum depression predisposes to maternal affective and somatic disorders. It is important to identify which women are at an increased risk of subsequent morbidity and would benefit from an intensified follow-up. Self-harm thoughts (SHTs), with or without other depressive symptomatology, might have prognostic value for maternal health beyond the postpartum period.

**Aim:**

This study is to investigate the somatic and psychiatric morbidity of postpartum women with SHTs, with or without other depressive symptoms, over a 7-year follow-up period.

**Materials and methods:**

The subjects for this study are derived from a population-based Swedish cohort of women who gave birth at Uppsala University Hospital (May 2006–June 2007) and who answered the Edinburgh Postnatal Depression Scale (EPDS) at 5 days, 6 weeks, and 6 months postpartum. Three groups were included: women reporting SHTs (SHT group, *n* = 107) on item 10 of the EPDS; women reporting depressive symptoms, i.e., EPDS ≥ 12 at 6 weeks and/or 6 months postpartum, without SHTs (DEP group, *n* = 94); and randomly selected controls screening negatively for postpartum depression (CTL group, *n* = 104). The number of diagnostic codes for somatic and psychiatric morbidity according to the International Statistical Classification of Diseases and Related Health Problems system, and the number of medical interventions were retrieved from medical records over 7 years following childbirth and were used as the outcome measures, together with any prescription of antidepressants and sick leave during the follow-up.

**Results:**

The SHT group had the highest psychiatric morbidity of all groups and more somatic morbidity than controls. Affective disorders were more common in the SHT and the DEP groups compared with controls, as well as antidepressant prescriptions and sick leave. One-fifth of women with SHTs did not screen positive for depressive symptoms; nevertheless, they had more somatic and psychiatric morbidity than the control group.

**Conclusion:**

Women reporting thoughts of self-harm in the postpartum period are at an increased risk of somatic and psychiatric morbidity during a follow-up of 7 years after delivery, and this increased risk may not be fully attributed to depressive symptoms. Results underline the importance of screening for self-harm symptoms postpartum and point to a need for individualized follow-up.

## Introduction

Postpartum depression (PPD) is a common and serious mental health disorder estimated to affect 8–15% of new mothers (1–3). The most recent fifth edition of the Diagnostic and Statistical Manual of Mental Disorders specifies the term peripartum depression as a major depressive episode with an onset during pregnancy or within 4 weeks after childbirth (4). Yet, in practice, the postpartum definition is extended to include the entire first year after delivery (1, 5–7). PPD remains an underdiagnosed condition, partly because some of the clinical manifestations, such as fatigue, weight, or sleep disturbance, are physiologically expected during the postpartum period (6, 8). Its consequences, however, may be severe. In the short term, PPD causes suffering for the affected woman and predisposes to disturbances in the mother–infant relationship. In a longer perspective, it is associated with subsequent maternal somatic and affective disorders as well as cognitive and behavioral developmental deficits of the child (7, 9). Furthermore, its most severe manifestation, suicide in the prolonged puerperium, is one of the leading causes of maternal death in developed countries (3, 10, 11).

Potentially long-term, severe PPD outcomes necessitate a prolonged follow-up for subsequent morbidity. However, it is important to identify which women are at an increased risk of depression recurrence and would actually benefit from an intensified follow-up. One of the especially worrying and severe expressions of PPD is the presence of self-harm thoughts (SHTs) or behaviors (3, 12–14). The prevalence of SHTs during pregnancy and postpartum varies among diverse populations, ranging from 5% (Finland) (15) to 15% (India) (16), whereas suicide accounts for 20% of deaths among postpartum women (3). Even though depression increases the risk for SHTs, suicidal behavior is not always accompanied by other depressive symptoms (17). Current screening efforts to identify women with PPD are based on the questionnaire tools, such as the Edinburgh Postnatal Depression Scale (EPDS) (18), that cover a broad spectrum of depressive symptoms including thoughts of self-harm (19).

To this end, the aim of this study is to investigate long-term somatic and psychiatric morbidity among women who reported thoughts of self-harm with or without depressive symptoms postpartum, compared to women with depressive symptoms without SHTs and to postpartum non-depressed women, over a 7-year follow-up period.

## Materials and Methods

### Study Population and Design

This study was performed as a part of the UPPSAT study, a large population-based longitudinal study of 2,318 women who gave birth at Uppsala University Hospital between May 2006 and June 2007. Participation rate in the UPPSAT study was 65%. All delivering women were asked to participate by receiving oral and written information about the study objectives; if a written consent was obtained, women were included in the study. Exclusion criteria from the UPPSAT were (1) women not being able to adequately communicate, write, or read in Swedish, (2) women whose personal data were kept confidential, and (3) women with intrauterine demise or infants immediately admitted to the neonatal intensive care unit. Moreover, women whose personal data were missing or who had moved to a different county were excluded from the study. The Regional Ethical Review Board in Uppsala approved the study protocol (UPPSAT, Dnr 2006/150).

### Study Groups

For this study, three groups of women were selected from the source population of the UPPSAT cohort. The first group (SHT) included all women who endorsed SHTs postpartum, based on the answer to EPDS item 10 (occurrence of thoughts of self-harm in the past 7 days). Possible answers were “never,” “hardly ever,” “sometimes,” and “quite often.” In total, 107 women answering other than “never” on this question at one or more of the three screening points postpartum (5 days, 6 weeks, and 6 months) were included in the SHT group.

The second study group (DEP) comprised 94 women who screened positively for depression (EPDS ≥ 12) at 6 weeks and/or at 6 months postpartum, but reported no SHTs (answering “never” to EPDS item 10) at 6 weeks or 6 months. The 5-day assessment point was not used for this purpose, since so early a time range does not correspond to the definition of PPD, but rather the more common “postpartum blues.”

The last study group consisted of 104 controls (CTL) who were randomly selected among participants who had screened negatively for depression (EPDS < 12) at 6 weeks and 6 months postpartum and reported no SHTs (answering “never” to EPDS item 10) at all three screening points postpartum.

In a subanalysis, we assessed the association of SHTs without other depressive symptoms with later morbidity. Therefore, the SHT group was further divided into women who screened positively for depression (EPDS ≥ 12) at any of the three postpartum screening points (*n* = 84) and those who consistently screened negatively (*n* = 23).

### Measures and Outcome Variables

Participants received self-administered structured questionnaires at 5 days, 6 weeks, and 6 months postpartum. These included the Swedish version of the EPDS, as well as a range of questions designed by the research team concerning age, educational level, work status, previous psychiatric contact, mood during pregnancy, hours of sleep postpartum, breastfeeding, and partner support. In the UPPSAT study, more variables were assessed, which were not included in this study.

The EPDS is a 10-item screening questionnaire for depression specifically addressed to postpartum women assessing symptoms over the past week (18). Total scores range from 0 to 30 and a cut-off of ≥12 points is advocated for PPD (20), with a sensitivity of 72% and a specificity of 88% (21). One of the EPDS items (item 10) addressed self-harm symptoms: “The thought of harming myself has occurred to me.”

The mothers’ medical records were investigated by one of the authors (HR) from the day of maternity hospital discharge up to October 15, 2013, regarding diagnoses related to somatic and psychiatric morbidity, reproduction, as well as health care interventions. The diagnoses were registered in the journals according to the International Statistical Classification of Diseases and Related Health Problems (ICD)-Coding System, 10th edition (22). ICD-10 is structured in major categories and subcategories including specific diagnoses. For example, the major category “Mental and behavioral disorders” includes several subcategories, such as “Mood [affective] disorders”, under which there are several specific diagnoses such as “Manic episode” or “Depressive episode.” In this study, the total number of specific diagnoses per participant related to somatic morbidity, psychiatric morbidity, and reproduction were recorded as indicators of overall morbidity.

Concerning somatic morbidity, diagnoses were grouped according to ICD-10 major categories. Major ICD categories (and their codes) included in somatic morbidity were: Certain infectious and parasitic diseases (A00-–B99); Neoplasms (C00–D48); Diseases of the blood and blood-forming organs and certain disorders involving the immune mechanism (D50–D89); Endocrine, nutritional and metabolic diseases (E00–E90); Mental and behavioral disorders (F00–F99); Diseases of the nervous system (G00–G99); Diseases of the eye and adnexa (H00–H59); Diseases of the ear and mastoid process (H60–H95); Diseases of the circulatory system (I00–I99); Diseases of the respiratory system (J00–J99); Diseases of the digestive system (K00–K93); Diseases of the skin and subcutaneous tissue (L00–L99); Diseases of the musculoskeletal system and connective tissue (M00–M99); Diseases of the genitourinary system (N00–N99); Pregnancy, childbirth, and the puerperium (O00–O99); Congenital malformations, deformations, and chromosomal abnormalities (Q00–Q99); Symptoms, signs, and abnormal clinical and laboratory findings not elsewhere classified (R00–R99); Injury, poisoning, and certain other consequences of external causes (S00–T98); External causes of morbidity and mortality (V01–Y98: mechanism of injury, e.g., transport accidents); and Factors influencing health status and contact with health services (Z00–Z99: intervention codes and descriptions of circumstances around contact). The study groups were compared according to the percentage of individuals with any specific diagnosis per major category.

Concerning psychiatric morbidity, in addition to the major category “Mental and behavioral disorders” (ICD codes F00–F99), diagnoses were also sorted into the underlying subcategories: “Organic, including symptomatic, mental disorders” (F00–F09), “Mental and behavioral disorders due to psychoactive substance use” (F10–F19), “Schizophrenia, schizotypal, and delusional disorders” (F20–F29); “Mood [affective] disorders” (F30–F39), “Neurotic, stress-related and somatoform disorders” (F40–F48); “Behavioral syndromes associated with physiological disturbances and physical factors” (F50–F59); and “Disorders of adult personality and behavior” (F60–F69). The study groups were compared according to the percentage of individuals with any specific diagnosis per subcategory. In addition, the prevalence of specific diagnoses under “Mood [affective] disorders” and “Neurotic, stress-related and somatoform disorders” during follow-up was calculated for the study groups and compared between groups.

Health care interventions were registered according to the Swedish designated coding system (23), referring to patient-directed interventions including medical examinations, diagnostic tests, surgical procedures, preventive care efforts, and treatments. The total number of health care interventions per participant and the number of *different* interventions per participant, registered during the follow-up period were recorded as an indicator of overall morbidity. The median number of interventions and of types of interventions were calculated for each study group, and comparisons were implemented. Furthermore, the number of interventions related to psychological support was specifically recorded, and study groups were accordingly compared.

Apart from the above diagnostic codes and health interventions, the medical records were also scrutinized regarding contact with the psychiatric care unit before the index childbirth, the number of childbirths and abortions during the follow-up period, sick leave (and whether the reason was due to somatic or psychiatric symptoms), and the prescription of antidepressants.

In a sub-analysis, to investigate the importance of SHTs that are independent of depression, differences in overall morbidity were investigated between SHT participants without depressive symptoms (*n* = 23) and the control group.

### Statistical Analyses

IBM SPSS Statistics version 22 was used for the analyses, and the significance level was set at *p* < 0.05. Comparisons were implemented across the three study groups as follows: for categorical variables, such as proportions of women with a certain diagnosis or intervention, Pearson Chi-square tests were used, with *post hoc* tests (Bonferroni corrected per specific outcome) when applicable. For the normally distributed scale variables, age, and follow-up time, one-way ANOVA was used. For non-normally distributed scale variables, such as the number of diagnoses and interventions in the study groups, Kruskal–Wallis tests were used, with *post hoc* pairwise Mann–Whitney *U* tests (Bonferroni corrected per specific outcome: three analyses, *p* < 0.017) when the overall test was significant.

## Results

### Descriptive and Postpartum Characteristics

Descriptive characteristics and self-reported postpartum variables for the index pregnancy in the three study groups (SHT, DEP, and CTL) are shown in Table 1. Women in the three groups did not differ regarding age, educational level, follow-up time, or number of childbirths or abortions during the follow-up.

**Table 1:** Descriptive information and self-reported variables for the index pregnancy for postpartum women with SHT, DEP, and CTLs.

Variable	SHT	DEP	CTL	Sig.
EPDS ≥ 12, 5 days postpartum (*n* = 271)	35 (43.2)	31 (36.0)	0 (0.0)	^a,b^
EPDS ≥ 12, 6 weeks postpartum (*n* = 286)	44 (50.0)	55 (58.5)	0 (0.0)	^a,b^
EPDS ≥ 12, 6 months postpartum (*n* = 278)	42 (52.5)	55 (58.5)	0 (0.0)	^a,b^
Age at first screening point (years), mean (SD), *n* = 305	30.5 (4.7)	30.4 (4.8)	31.7 (3.4)	ns
No college or university (*n* = 284)	59 (67.8)	52 (55.9)	71 (68.3)	ns
Unemployed/student/sick leave at baseline (*n* = 286)	18 (20.2)	24 (25.8)	11 (10.6)	^b^
Previous psychiatric contact, self-reported (*n* = 286)	48 (53.9)	38 (40.9)	19 (18.3)	^a,b^
Previous psychiatric contact, medical record (*n* = 305)	36 (33.6)	23 (24.5)	7 (6.7)	^a,b^
Lowered mood during pregnancy (*n* = 282)	51 (58.6)	39 (41.9)	14 (13.7)	^a,b^
Elective cesarean section (*n* = 305)	14 (13.1)	3 (3.2)	4 (3.8)	^a,c^
Sleep < 6 h/night at 6 weeks postpartum (*n* = 285)	35 (39.3)	36 (38.7)	18 (17.5)	^a,b^
Sleep < 6 h/night at 6 months postpartum (*n* = 277)	20 (25.3)	16 (17.0)	15 (14.4)	ns
No/non-exclusive breastfeeding, 6 weeks postpartum (*n* = 286)	23 (25.8)	22 (23.7)	12 (11.5)	^a^
No breastfeeding 6 months postpartum (*n* = 267)	24 (30.4)	27 (30.0)	17 (17.3)	ns
Suboptimal help from partner, postpartum (*n* = 261)	43 (65.2)	64 (69.6)	47 (45.6)	^a,b^
Follow-up time (weeks), mean (SD), *n* = 305	360.6 (15.5)	360.4 (14.0)	357.5 (15.9)	ns
Underwent ≥ 1 delivery during follow-up time (*n* = 305)	51 (47.7)	51 (54.3)	53 (51.0)	ns
Underwent ≥ 1 abortion during follow-up time (*n* = 305)	13 (12.1)	8 (8.5)	8 (7.7)	ns

*Values are *n* (%) if not otherwise specified*.

*CTL, healthy controls; DEP, depressive symptoms without self-harm thoughts; EPDS, Edinburgh Postnatal Depression Scale; ns, no difference; SHT, self-harm thought*.

*Pearson Chi-square tests for categorical variables, with *post hoc* tests given overall significance (Bonferroni corrected within specific outcome) for categorical variables and one-way ANOVA for continuous variables*.

*Significance (*p* < 0.05): ^a^difference between SHT and CTL group; ^b^difference between DEP and CTL group; and ^c^difference between SHT and DEP group*.

Concerning the index pregnancy, the SHT and the DEP groups were more depressed during pregnancy and more often had contact with psychiatric health care, whether self-reported or based on medical records, compared to controls. Within the SHT group, the proportion of women screening positively for depression was approximately equal at the three screening points. Participants with SHTs were more likely to have delivered *via* elective cesarean section compared to both the DEP and the control group. Furthermore, in comparison with controls, women in the SHT and DEP groups reported poorer support from their partner, whereas women in the SHT group were also less likely to breastfeed at 6 weeks postpartum.

### Follow-Up Morbidity

Overall morbidity in the three study groups is presented in Table 2. Consistently, both the SHT and the DEP groups showed higher morbidity than controls and were more often on sick leave. Furthermore, the reason for sick leave noted in the journals differed: SHT group women were more often ascribed a combined somatic and psychiatric reason, while controls had more often solely a somatic reason. Depression alone was not a better marker for any of the overall morbidity measures compared with SHTs; on the contrary, a trend toward a higher number of psychiatric diagnoses in the SHT group compared with the DEP group reached baseline statistical significance (*p* < 0.05) but not the Bonferroni corrected level of *p* < 0.017.

**Table 2:** Overall morbidity during the follow-up period for postpartum women with SHT, DEP, and CTLs.

Morbidity variable	SHT	DEP	CTL	Sig.
Total number of ICD-10 diagnoses related to somatic morbidity, median (IQR)	12 (17)	10.5 (19)	7 (8)	^a,b^
Total number of ICD-10 diagnoses related to psychiatric morbidity, median (IQR)	1 (4)	0 (2)	0 (0)	^a,b^
Number of different health interventions, median (IQR)	8 (11)	8 (9)	6 (6)	^a,b^
Total number of health interventions, median (IQR)	11 (18)	12.5 (19)	7 (9)	^a,b^
Sick leave, *n* (%)	59 (55.1)	56 (59.6)	34 (32.7)	^a,b^
Somatic reason, *n* (% of Sick leave)	33 (55.9)	34 (60.7)	24 (70.6)	ns
Psychiatric reason, *n* (% of Sick leave)	9 (15.3)	10 (17.9)	5 (14.7)	ns
Both somatic and psychiatric reason, *n* (% of Sick leave)	17 (28.8)	12 (21.4)	5 (14.7)	^a^

*CTL, healthy controls; DEP, depressive symptoms without self-harm thoughts; ICD, International Classification of Diseases; IQR, interquartile range; ns, no difference; SHT, self-harm thought*.

*Kruskal–Wallis test for continuous variables, with *post hoc* Mann–Whitney *U* tests given overall significance (Bonferroni corrected within specific outcome), and Pearson Chi-square tests for categorical variables, with *post hoc* tests given overall significance (Bonferroni corrected within specific outcome)*.

*Significance (*p* < 0.05): ^a^difference between SHT and CTL groups; ^b^difference between DEP and CTL group; ^c^difference between SHT and DEP group*.

Somatic disorders of the respiratory system, digestive system, skin, musculoskeletal system and connective tissue, as well as symptoms with no diagnosis, and codes for interventions and special circumstances for the contact were more common in the SHT group compared to controls (Table 3). Notably, any differences between the DEP group and controls were not statistically significant.

**Table 3:** Somatic morbidity as registered in the medical journal during the follow-up period.

ICD major category	ICD codes	SHT	DEP	CTL	Sig.
Diseases of the respiratory system	I00–I99	73 (68.2%)	60 (63.8%)	51 (49.0%)	^a^
Diseases of the digestive system	K00–K93	38 (35.5%)	25 (26.6%)	17 (16.3%)	^a^
Diseases of the skin and subcutaneous tissue	L00–L99	47 (43.9%)	35 (37.2%)	23 (22.1%)	^a^
Diseases of the musculoskeletal system and connective tissue	M00–M99	59 (55.1%)	39 (41.5%)	38 (36.5%)	^a^
Symptoms, signs, and abnormal clinical and laboratory findings not elsewhere classified	R00–R99	75 (70.1%)	55 (58.5%)	56 (53.8%)	^a^
Factors influencing health status and contact with health services	Z00–Z99	80 (74.8%)	63 (67.0%)	61 (58.7%)	^a^

*Persons with at least one diagnosis within each major category, in SHT, DEP, and CTL groups*.

*CTL, healthy controls; DEP, depressive symptoms without self-harm thoughts; ICD, International Classification of Diseases; SHT, self-harm thought*.

*Pearson Chi-square tests, with *post hoc* tests given overall significance (Bonferroni corrected within specific outcome)*.

*Significance (*p* < 0.05): ^a^difference between SHT and CTL groups; ^b^difference between DEP and CTL groups; ^c^difference between SHT and DEP groups*.

*Some included ICD major categories are omitted (no differences): Certain infectious and parasitic diseases (A00–B99); Neoplasms (C00–D48); Diseases of the blood and blood-forming organs and certain disorders involving the immune mechanism (D50–D89); Endocrine, nutritional and metabolic diseases (E00–E90); Diseases of the nervous system (G00–G99); Diseases of the eye and adnexa (H00–H59); Diseases of the ear and mastoid process (H60–H95); Diseases of the circulatory system (I00–I99); Diseases of the genitourinary system (N00–N99); Pregnancy, childbirth, and the puerperium (O00–O99); Congenital malformations, deformations, and chromosomal abnormalities (Q00–Q99); Injury, poisoning, and certain other consequences of external causes (S00–T98); and External causes of morbidity and mortality (V01–Y98)*.

Outcomes related to psychiatric health are displayed in Table 4. The number of women with any psychiatric diagnosis during the follow-up was higher in the DEP group compared to controls and was highest among SHT women. Women in the SHT and DEP groups were more often diagnosed with an affective disorder and were more likely to receive a prescription of antidepressants and supportive sessions within health care. Furthermore, women with SHTs, with or without depressive symptoms, were more often ascribed a diagnosis within “Neurotic, stress-related and somatoform disorders” than controls, as well as the specific diagnoses of “depressive episode” or “recurrent depressive disorder.”

**Table 4:** Psychiatric morbidity as registered in the medical journal during the follow-up period.

Morbidity variable	ICD code(s)	SHT	DEP	CTL	Sig.
**ICD major category, any diagnosis, *n* (%)**					
Mental and behavioral disorders	F00–F99	58 (54.2%)	34 (36.2%)	20 (19.2%)	^a,b,c^

**Subcategories, any diagnosis, *n* (%)**					
Mood (affective) disorders	F30–F39	32 (29.9%)	20 (21.3%)	9 (8.7%)	^a,b^
Depressive episodes	F32	28 (26.2%)	17 (18.1%)	8 (7.7%)	^a^
Recurrent depressive disorder	F33	11 (10.3%)	7 (7.4%)	2 (1.9%)	^a^
Neurotic, stress-related and somatoform disorders	F40–F48	38 (35.5%)	25 (26.6%)	14 (13.5%)	^a^
Phobic anxiety disorders	F40	5 (4.7%)	2 (2.1%)	0 (0.0%)	ns
Other anxiety disorders	F41	28 (26.2%)	20 (21.3%)	6 (5.8%)	^a,b^
Reaction to severe stress, and adjustment disorders	F43	17 (15.9%)	12 (12.8%)	8 (7.7%)	ns

**Interventions, ≥1, *n* (%)**					
Prescription of antidepressants	–	49 (45.8%)	31 (33.0%)	8 (7.7%)	^a,b^
Supportive session with health care professional	–	47 (43.9%)	38 (40.4%)	15 (14.4%)	^a,b^

*Persons with any diagnosis within each category; persons with a specific diagnosis; and persons receiving certain interventions in SHT, DEP, and CTL groups*.

*CTL, healthy controls; DEP, depressive symptoms without self-harm thoughts; ICD, International Classification of Diseases; SHT, self-harm thought*.

*Pearson Chi-square tests, with *post hoc* tests given overall significance (Bonferroni corrected within specific outcome)*.

*Significance (*p* < 0.05): ^a^difference between SHT and CTL groups; ^b^difference between DEP and CTL groups; and ^c^difference between SHT and DEP groups; (ns) no difference*.

*Some ICD subcategories are omitted (no differences, few cases): Organic, including symptomatic, mental disorders (F00–F09); Mental and behavioral disorders due to psychoactive substance use (F10–F19); Schizophrenia, schizotypal, and delusional disorders (F20–F29); Behavioral syndromes associated with physiological disturbances and physical factors (F50–F59); and Disorders of adult personality and behavior (F60–F69)*.

*Some specific ICD diagnoses are omitted (no differences, with *n* ≤ 3 cases in each group): Manic episode (F30), Bipolar affective disorder (F31), Persistent mood [affective] disorders (F34), Other mood [affective] disorders (F38), Unspecified mood [affective] disorder (F39), Phobic anxiety disorders (F40), Obsessive-compulsive disorder (F42), Reaction to severe stress, and adjustment disorders (F43), Dissociative [conversion] disorders (F44), Somatoform disorders (F45), and Other neurotic disorders (F48)*.

Finally, follow-up morbidity was assessed among women in the SHT group without depressive symptoms. There were 21.5% of women with SHTs who were screened negatively for depressive symptoms postpartum. Comparisons of overall morbidity between this subgroup of SHT subjects and control women are shown in Table 5. Non-depressed women in the SHT group received more somatic and/or psychiatric diagnoses than controls, as well as a larger number of different health interventions.

**Table 5:** Subanalysis of overall morbidity during the follow-up period for those with SHT screening negatively for depression compared to CTLs.

Morbidity variable	SHT without depressive symptoms	CTL	Sig.
Total number of somatic ICD codes, median (IQR)	11 (16)	7 (8)	*
Total number of psychiatric ICD codes, median (IQR)	1 (2)	0 (0)	*
Number of different health interventions, median (IQR)	9 (10)	6 (6)	*
Total number of health interventions, median (IQR)	10 (18)	7 (9)	ns
Sick leave, *n* (%)	12 (52.2%)	34 (32.7%)	ns
Somatic reason, *n* (% of sick leave)	7 (58.3%)	24 (70.6%)	ns
Psychiatric reason, *n* (% of sick leave)	3 (25.0%)	5 (14.7%)	ns
Both somatic and psychiatric reason, *n* (% of sick leave)	2 (16.7%)	5 (14.7%)	ns

*CTL, healthy controls; DEP, depressive symptoms without self-harm thoughts; ICD, International Classification of Diseases; IQR, interquartile range; ns, no difference; SHT, self-harm thought*.

*Mann–Whitney U test for continuous variables and Pearson Chi-square tests for categorical variables*.

Significance: *p < 0.05.

## Discussion

This study found a higher psychiatric morbidity over a 7-year follow-up period after childbirth in a population-based sample of women expressing SHTs in the first 6 months postpartum, with or without depressive symptoms (SHT group), compared with women screening positively for PPD while reporting no SHTs (DEP group) and control women reporting neither SHTs nor depressive symptoms. Compared to controls, SHT women also showed a higher burden of somatic disease. In both, the SHT and the DEP groups, in comparison with controls, affective disorders, prescription of antidepressants, and sick leave, were more common during follow-up. More than one-fourth of the women who endorsed thoughts of self-harm postpartum, with or without depressive symptoms, were ascribed in their medical records the diagnosis of “depressive episode” at least once during follow-up.

Several studies have previously examined postpartum depressed women’s well-being, compared to non-depressed women, as well as regarding behavioral outcomes of their children several years after childbirth (24, 25). To our knowledge, however, this is the first study prospectively investigating both physical and psychiatric morbidity, assessed *via* medical records, in women with SHTs postpartum and/or depressive symptoms. It is well established that women with PPD are at higher risk of subsequent depression compared to non-depressed women (9, 25–27). It has been suggested that PPD actually comprises two different subgroups: first-time depression that indicates a vulnerability to childbirth; and recurrent, chronic mood disorder, with an increased risk of subsequent episodes (26). In the same context, previous studies support the notion that it might be the recurrence of depressive episodes, rather than the postpartum depressive episode *per se*, which are related to problems with the child’s cognitive and behavioral development (9, 25–27). A Swedish study further shows that PPD also increases the risk of somatic illness (27).

The origins of SHTs were not addressed by this project. However, some background variables were included. Women in the SHT and DEP groups had more often a history of earlier psychiatric contact than controls; previous psychiatric morbidity is a known risk factor for both PPD (28–31) and SHTs (32). Similarly, in line with previous research, depressive symptoms postpartum were associated with depressed mood during pregnancy and poor partner support (28–31). Women in the SHT group were also seen to breastfeed less at 6 weeks after delivery. Besides being a possible sign of depression or anxiety *per se* (33), non-breastfeeding also predisposes for later depression (34), possibly due to not taking advantage of its potentially calming effects.

The overall morbidity was higher in the SHT and the DEP groups versus controls. However, concerning specific categories of somatic disease, there were differences between the SHT group and controls, but not between the DEP group and controls. It might be that the difference was smaller in the latter comparison and that it would be detectable with a larger sample size. The pertinent finding, however, was the increased somatic and psychiatric disease burden among women with SHTs compared to non-depressed controls. Even among SHT women without depressive symptoms, overall measures of morbidity in terms of total number of diagnoses were higher than in the healthy population.

Consistently with previous literature, these results support an increased risk of subsequent depression among women with PPD (35–37). Most importantly, the present results indicate that women with both SHTs and depressive symptoms postpartum might be at even higher risk. Interestingly, more than one-fifth of women with SHTs scored low on EPDS, indicating no overall depressive symptomatology; and at baseline, the SHT group on average did not have more symptoms of depression than the DEP group. This stands against the idea that SHTs were merely a sign of worse depression. On the contrary, our results indicate that SHTs may be related to other factors, such as anxiety or emotional regulation. For example, women in the SHT group were more likely to have delivered *via* elective cesarean section. Besides several somatic indications, a reason behind elective cesareans is when women experience severe anxiety related to childbirth (38). Nonetheless, it cannot be ruled out that the presence of SHTs not always accompanied by concurrent depressive episodes might depend on an inherent weakness of the EPDS as a diagnostic tool. Despite that fact, such an inherent limitation of the test could not account for all differences between the groups observed in the present study. Evidently, it could be presumed that the overall psychiatric background of women with SHTs is explanatory of the increased morbidity burden instead of the postpartum symptoms. However, irrespective of any causal or unconfounded relationships, if the mere self-report of self-harm symptoms is as a marker for subsequent morbidity, it may still be used to identify women who would benefit from individualized follow-up.

The increased somatic morbidity of women reporting SHTs could on the one hand be interpreted in the context of physical symptoms often accompanying depression (39). It is also well known that depression increases the risk, or accelerates the onset, of chronic somatic diseases like cardiovascular disease, diabetes, and obesity (40–43). The underlying mechanisms are largely unknown, albeit suspected to include metabolic, immune-inflammatory, autonomic, and hypothalamus-pituitary-adrenal axis dysregulation (44). Somatic symptoms, such as back pain, abdominal pain, or headache, are the leading cause of outpatient medical visits and also the predominant reason why patients with common mental disorders such as depression and anxiety initially present in the medical care setting (39, 45, 46). Notably, one of the coding categories of somatic disease related to earlier SHTs consists of symptoms which do not fit with any diagnosis. One might consider several interpretations. First, it could be that women with SHTs are more prone to somatization. Sensitivity to, and distress around, unusual body sensations is related to anxiety (47) and certain personality traits such as neuroticism (48). If this is the case, a major interpretation of this study is that postpartum SHTs predispose for a higher health care consumption, as well as for the risk of being overdiagnosed with somatic and psychiatric disorders, rendering women at risk for unnecessary interventions. Second, health care professionals might not perform as well at diagnosing these women or are prone to interpret them differently than other patients. Third, there might be a common pathology underlying both SHTs and somatic symptoms that is yet to be revealed.

### Strengths and Limitations

The strengths of this study entail the large population-based sample with several available variables on an individual basis and the participation rate of above 60% (49), which could be considered as high in a population-based setting. However, as a previous Swedish study has suggested, the dropout could be associated with vulnerability factors such as lower income, lower education, non-Nordic origin, living without a partner, and previous psychiatric diagnosis (50). Therefore, an underestimation of the total burden of PPD, and thoughts of self-harm postpartum cannot be ruled out. The use of the self-administered EPDS, instead of a psychiatric interview, also comprises a methodological limitation even though the scale has been validated in the Swedish population (51). Similarly, SHTs were not assessed *via* a validated method, but *via* a single question from the EPDS that might have caused misclassification. Nevertheless, the prevalence of depression (11.1%) and thoughts of self-harm (6.2%) in the postpartum period in the UPPSAT study are in agreement with previous reports (1, 3, 52), as is also the high prevalence of depression among those reporting SHTs (78.5%) (3, 12–14).

The analyses included multiple outcomes. Since we did not correct for the total number of tests, it might be that some of the significant results are chance findings. For all *post hoc* tests, we performed Bonferroni correction for each specific outcome, which may have lowered the risk of such type I error all the while potentially leading to type II errors. However, the results consistently point toward higher morbidity among women expressing SHTs than in a non-depressive population and possibly higher morbidity than in depressed individuals without SHTs. These results need to be replicated in a larger sample, especially since several of the outcomes had very low prevalence.

Limitations linked to the design of the study should also be taken into account. During 2006–2007, a shift in the documentation system (from scanned journal documents to electronic-based journal system) used by health care professionals took place in Uppsala County, which may have led to some missed diagnoses. Due to this shift, it was also not possible to investigate women’s morbidity prior to childbirth. Furthermore, the lack of access to private health care records, as well as the records of women who only recently moved to the study site, could have led to an underestimation of previous psychiatric morbidity.

### Clinical Significance

The vulnerability of women with thoughts of self-harm for subsequent psychiatric and somatic health problems stresses the importance of screening for this symptom in the postpartum period, especially among women with depression. Even the self-report of SHTs *via* a single question of the EPDS questionnaire during postpartum was found to be associated with subsequent morbidity, also among women without depression, indicating the prognostic value of this item. This is important considering that a high proportion of women screening negatively for depression also have a high risk for subsequent illnesses. However, this study cannot conclude on whether an intervention, like intensive psychiatric follow-up, would affect rates of subsequent morbidity. Therefore, future studies should address this issue.

### Conclusion

This study found that women reporting thoughts of self-harm in the postpartum period were at an increased risk of psychiatric and somatic morbidity during a follow-up period of 7 years after delivery, and this increased risk may not be fully attributed to overall depressive symptoms. Therefore, screening for SHTs in the postpartum period could provide a window for support and treatment aiming to prevent future morbidity.

## Ethics Statement

This study was carried out in accordance with the recommendations of the Uppsala Regional Ethical Review Board with written informed consent from all subjects. All subjects gave written informed consent in accordance with the Declaration of Helsinki. The protocol was approved by the Regional Ethical Review Board in Uppsala (UPPSAT, Dnr 2006/150).

## Author Contributions

Conceptualization: AS and FP. Acquisition of data: AS, HR, and FP. Analysis and interpretation of data: SI, AS, HR, MG, CA, and FP. Initial drafting of the manuscript: HR and MG. Critical revision of the manuscript for intellectual content: SI, AS, CA, and FP. Final approval of the version to be published: SI, AS, HR, MG, CA, and FP.

## Conflict of Interest Statement

The authors declare that the research was conducted in the absence of any commercial or financial relationships that could be construed as a potential conflict of interest.
